# Experimental Characterization of Spurious Signals in Magnetic Nanoparticles Enhanced Microwave Imaging of Cancer

**DOI:** 10.3390/s21082820

**Published:** 2021-04-16

**Authors:** Ovidio M. Bucci, Gennaro Bellizzi, Sandra Costanzo, Lorenzo Crocco, Giuseppe Di Massa, Rosa Scapaticci

**Affiliations:** 1 Department of Electric Engineering and Information Technologies, University of Naples Federico II, 80125 Naples, Italy; bucci@unina.it; 2 Institute for the Electromagnetic Sensing of the Environment, National Research Council of Italy, 80124 Naples, Italy; crocco.l@irea.cnr.it (L.C.); scapaticci.r@irea.cnr.it (R.S.); 3 National Interuniversity Consortium for Telecommunications, 43124 Parma, Italy; 4 Department of Computer, Modeling, Electronics and Systems Engineering, University of Calabria, 87036 Rende, Italy; costanzo@dimes.unical.it (S.C.); giuseppe.dimassa@unical.it (G.D.M.)

**Keywords:** instrumental drift, magnetic nanoparticles, microwave imaging, scattering parameters, spurious magnetic effects

## Abstract

Magnetic nanoparticles enhanced microwave imaging relies on the capability of modulating the response of such nanocomponents at microwaves by means of a (low frequency) polarizing magnetic field. In medical imaging, this capability allows for the detection and imaging of tumors loaded with nanoparticles. As the useful signal is the one which arises from nanoparticles, it is crucial to remove sources of undesired disturbance to enable the diagnosis of early-stage tumors. In particular, spurious signals arise from instrumental drift, as well as from the unavoidable interaction between the polarizing field and the imaging system. In this paper, we experimentally assess and characterize such spurious effects in order to set the optimal working conditions for magnetic nanoparticles enhanced microwave imaging of cancer. To this end, simple test devices, which include all components typically comprised in a microwave imaging system, have been realized and exploited. The experiment’s results allow us to derive design formulas and guidelines useful for limiting the impact of unwanted magnetic effects, as well as that relative to the instrumental drift on the signal generated by the magnetic nanoparticles-loaded tumor.

## 1. Introduction

Magnetic Nanoparticles (MNPs)-enhanced MicroWave Imaging (MWI) is an emerging cancer diagnostic modality [1], wherein a tumor’s detection and imaging is achieved by exploiting MNPs as modulable contrast agents selectively delivered to the tumor [2]. The concept is very simple and it relies on the possibility of switching off the response of the MNPs at microwaves by applying a static (or quasi-static) Polarizing Magnetic Field (PMF) of proper intensity [3,4]. Accordingly, by switching the PMF on and off, one can acquire two different datasets of the scenario under test: in the first one, in the absence of the PMF, the MNPs-loaded tumor responds to the impinging microwave stimulus; in the second one, the presence of the PMF quenches the MNPs response. These two datasets only differ for the response of the MNPs accumulated in the tumor, since MNPs-free tissue surrounding the tumor is not affected by the PMF. Hence, the difference of these data singles out the response of the MNPs-targeted tumor, enabling the straightforward and highly selective detection and imaging of the tumor. This circumstance is particularly appealing in the case of early-stage tumors, whose diagnosis through standard MWI is impaired by the relatively low intrinsic electric contrast existing between cancerous and healthy tissue [5,6]. So far, this novel diagnostic concept has been assessed through accurate and realistic numerical simulations [7,8], as well as through experiments on simplified breast phantoms and measurement setups [9,10,11,12].

The use of MNPs as contrast agents in MWI of cancer is clinically viable due to their biocompatibility (thanks to which they have already been approved for clinical use [13]), and the possibility of their selective accumulation in the cancer cells via active targeting [14]. In particular, the latter feature is achieved by functionalizing the MNPs with biomolecules that have high affinity with the receptors typically overexpressed by cancer cells. Indeed, this favors their selective binding to the cell membrane and subsequent internalization. In this way, MNPs mainly accumulate into the cancer tissue once injected, and spread out throughout the body via the bloodstream. Of course, ensuring a sufficient uptake of MNPs is crucial for diagnostic purposes, and technological improvements are ongoing due to their widespread adoption in medical applications.

From a critical analysis of the results available in the literature (see [11]), it turns out that a realistic amount of MNPs deliverable to the tumor via active targeting is around 3.5 mg per 1 cm of tumor size. This is a very low amount, translating into a very low useful signal, which could be challenging to detect with standard MWI systems due to the unavoidable presence of undesired disturbances on the measured data. In particular, such disturbances arise from measurement noise and instrumental drift, whose dynamics can be comparable to, or even larger than that of, the useful signal. Therefore, in view of the application of MNPs as modulable contrast agents in MWI of early-stage tumors, a fundamental step is to assure the attainment of such detection capability.

Instrumental drift represents the main source of disturbance, as highlighted in [10]. Therein, it has been experimentally shown that drift is a very slowly time-varying signal, with an effective spectral component (i.e., above the noise level) not larger than some tens of Hz. Hence, its masking effect on the useful signal can be counteracted by adopting a sinusoidal PMF modulation (in place of an “on-off” modulation), with a frequency higher than the effective drift bandwidth. Incidentally, adopting a sinusoidal modulation also makes the recovery of the useful signal easy by means of a simple coherent filtering of the measured signal (in place of making the difference between the “on” and the “off” data acquired in two different measurements [2]). Moreover, the noise level, in turn, can be reduced by increasing measurement time, namely the number of acquired measurement samples.

On the basis of these outcomes in [11], we have estimated, for different kinds of breast phantom, the detection limits (i.e., the minimum amount of MNPs actually detectable) achievable by exploiting a sinusoidal PMF modulation (whose frequency of 5 Hz was set according to the spectral content of the drift characterizing the system in [11]), in order to establish the capability of the approach for the diagnosis of early-stage breast tumors.

The experiments in [11] have shown that, for the measurement setup adopted, our approach is able to detect an amount of MNPs as low as 2–7 mg, depending on the phantom, which is compatible with the amount of 3.5 mg of MNPs mentioned above. Moreover, based on the experimental results on the microwave magnetic response of MNPs in a PMF [4], in [11], it has been estimated that the above limits can be immediately halved (thus approaching the threshold of 3.5 mg) by simply doubling the PMF amplitude, i.e., by exploiting a more powerful PMF generator. However, the study also highlighted that a further lowering of the detection limit by employing better measurement instrumentation (i.e., characterized by a lower drift or noise level) is not possible due to the presence of a “spurious” modulated signal (i.e., detected in absence of MNPs in the phantoms) indistinguishable from that yielded by the MNPs-loaded tumor.

As discussed in [11], two mechanisms can be responsible for the observed spurious signals. The first one is the force exerted by the PMF on the microwave currents flowing in the conductors of the MWI system and in the phantom, which induce a modulation of the useful signal at the same frequency of the applied PMF. The second mechanism is related to the possible presence of hidden magnetic materials in the MWI system, which respond to the applied PMF in the same way as the MNPs, i.e., by producing a modulated signal at a frequency which is twice the frequency of the modulated PMF. Now, while the first signal can be easily filtered out (if significant), since it does not overlap with the useful spectral component, the presence of hidden magnetic materials is critical, as their effect cannot be separated from the useful signal, but only, at most, attenuated. This limits the possibility of detecting tumors smaller than 1 cm, which is the actual goal of the diagnostic approach. Hence, an important step is to reduce, as much as is possible, spurious effects due to magnetic materials, as they could mask the MNPs response and impair the detection of a tumor.

The aim of this work is to experimentally characterize the spurious magnetic effects yielded by the interaction of the PMF with materials and components usually present in an MWI system for cancer diagnosis. Moreover, the influence of the instrumental drift, which represents the other main source of disturbance for the application at hand, has been further analyzed and characterized.

It is worth noting that the nonmagnetic nature of the materials typically employed in phantoms to mimic human tissues, and their relatively low conductivity as compared to that of the metallic parts of the MWI system, allows one to exclude that an appreciable spurious signal can arise from these materials. As a result, one can exclude the phantom from such a characterization. Moreover, since the spurious signals due to each component/part of the MW system, in practice, do not affect each other (being the mutual interactions negligible) one can characterize the single components/parts separately (i.e., not necessarily as a whole) by, for instance, including them in a test device (TD) specifically meant for this purpose. The advantage of this is that such an ad-hoc TD can be more versatile and simple, and enable the characterization of the response of a single component at a time.

According to the above, TDs consisting of properly designed strip-lines have been considered. These TDs provide a convenient study environment, as not only can they be carefully modeled from an electromagnetic point of view, but they also include all the materials and components typically present in an MW radiating system. As such, they allow us to achieve quite general results while being simple.

By exploiting the results obtained from studying the adopted TDs, design formulas and guidelines, to address the choice of PMF modulation (amplitude and frequency) and the type of measurement (transmission or reflection), have been derived. These guidelines allow us to limit the impact of spurious magnetic effects and drift on the useful signal generated by an MNPs-loaded target, and to improve the detection limits.

The paper is organized as follows. In Section 2, the adopted TDs, their electromagnetic model, as well as the implemented devices and the entire measurement set-up adopted for the characterization are presented. In Section 3, the results of the experimental characterization are presented and discussed. The design formulas and guidelines are derived in Section 4. Their application to the case of MNPs-enhanced MWI of breast cancer is discussed in the same section. Conclusions follow in Section 5. Part of the mathematical derivation is included in the Appendix A.

## 2. Materials and Methods

### 2.1. Choice of the Test Devices

As already noted, there are essentially two possible sources of spurious signals due to the application of a PMF on an MW device.

The first disturbance arises from the action exerted by the PMF on the microwave currents flowing in the conducting parts of the system. As it happens in magneto-plasmas [15], the PMF modifies the relationship between the current density and the electric field, leading to a change in medium conductivity (which becomes anisotropic). The magnitude of such modification increases with the mobility of the charge carriers. Since the mobility of the electrons in metals is much higher than that of ions in aqueous solution, it turns out that the most critical components are the metallic parts of the MW system, and that possible contributions arising from the currents induced in phantoms can be confidently neglected as compared to the first ones.

Therefore, to investigate this effect, the adopted TD was a strip line (hereafter, *strip-1*). Such a simple device is mostly made of a printed metallic surface (of size comparable to the wavelength) and therefore represents the worst case in terms of the effect under test. Moreover, it is quite versatile (as it allows one to apply the PMF in different ways), easy and cheap to manufacture, completely shielded from externa disturbance, and its MW behavior is well described from a theoretical (i.e., analytical) standpoint.

The second source of spurious magnetic effects was the possible presence of hidden magnetic materials in the MW systems, whose magnetization under an applied PMF can perturb the MW response. For instance, this could be the case of tin solders, ubiquitously present in MW systems, or of commercially available adapters/connectors, which often present a thin layer of nickel below the surface, useful for favoring the adhesion of gold plating (obviously, the presence of magnetic materials is to be excluded in phantoms mimicking the human body). In particular, the generation of spurious magnetic effects by connectors was qualitatively assessed in [16].

To quantitatively investigate this second effect, we adopted as TDs two strip lines (hereafter, *strip-2* and *strip-3*) which had the same dimensions as *strip-1*, but which were cut in the middle in order to insert the MW component to be tested between the two pieces of the strips. In particular, a tin welding and two connectors joined by a trough were tested. It is worth noting that we inserted the components to be tested at the center of the strip lines to have symmetrical TDs as similar to each other, with the components under test subject to a known, uniform PMF.

The spurious magnetic effects were estimated by applying the PMF on the central part of each TD and measuring the variation of the scattering parameters (SPs) through a Vector Network Analyzer (VNA). To this end, each TD was terminated with two strip line-sma adapters (welded to the strip) plugged to the VNA through two coaxial cables. All the strips were sufficiently long so that one could confidently neglect the fringing PMF acting on the terminal connectors. In this way, one could get rid of possible spurious magnetic effects arising from connectors, which would otherwise have overlapped with those generated by the load under test, thus impairing the quantitative assessment.

Finally, let us note that the adopted strip lines were designed to have a characteristic impedance of 50 Ω, namely equal to that of the employed VNA, connectors/adapters and coaxial cables. This assured that each TD was well matched and exhibited low reflection coefficients and high transmission ones, so that one could simultaneously analyze the spurious effects in the two opposite situations of low and high SPs.

### 2.2. Manufactured Test Devices

**Figure 1:**
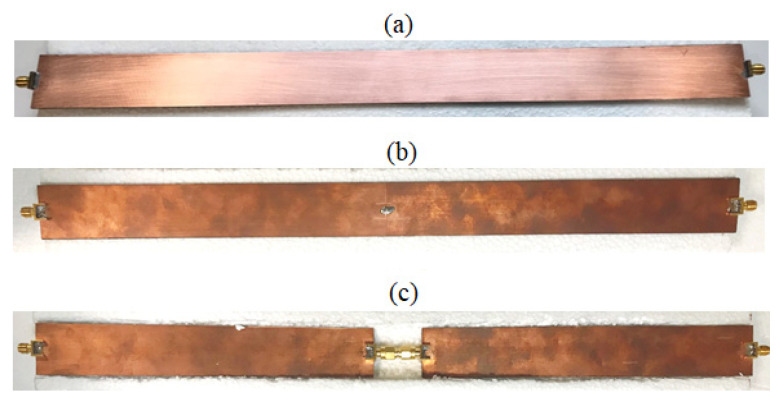
Pictures of the test devices (TDs): (**a**) *strip-1*; (**b**) *strip-2*; (**c**) *strip-3*.

Figure 1 shows the three TDs designed and realized (at the Microwave Laboratory of the University of Calabria) for the characterization at hand.

As shown in Figure 1a, *strip-1* was a simple (i.e., homogeneous) strip-line with no central load, and it was the same as that preliminarily tested in [16], namely a copper strip of a width of *w_S_* = 1.2 mm, crammed between two dielectric layers, of a thickness of *h* = 0.76 mm, and of a dielectric constant of *ε_s_* = 2.33, in turn covered by two copper ground planes, of a width of *w_g_* = 30 mm. The strip-line length was *l_S_* = 390 mm, while the overall length, including the terminal sma connector-strip line adapters, was equal to 410 mm.

*Strip-2* consisted of two half strip-lines, having an equal length of *l_S_*/2 = 195 mm, and soldered together by a tin welding representing the central load, and ended by two sma connectors-strip line adapters, as shown in Figure 1b. The overall length was still equal to 410 mm.

Finally, *strip-3* was similar to *strip-2*, but the two half strip lines were connected at the center by two additional sma connectors and a through connector, as shown in Figure 1c, representing the central load. The overall central connector had a length of 30 mm so that the overall length of *strip-3* was 440 mm.

### 2.3. Conceptual Scheme of the Test Devices

**Figure 2:**
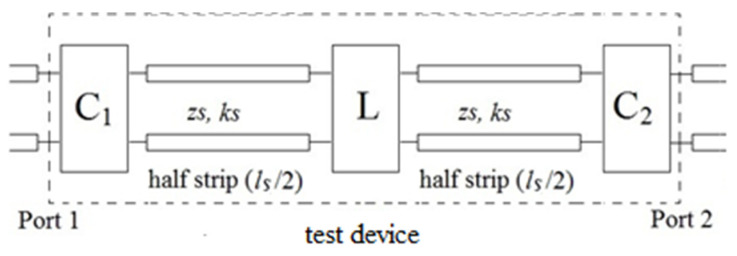
Scheme of the TDs.

In the absence of a PMF, the three TDs could be schematized as shown in Figure 2. The transmission lines represent the two parts of the strip-line, each of length *l_S_*/2, on the left and right of the central load, with a characteristic impedance of *z_s_*≈*z*_0_ = 50 Ω (being z_0_ the characteristic impedance of the VNA), and a propagation constant of *k_s_* = 2π*f*√*ε_s_*/*c*, where *ε_s_* is the relative permittivity of the dielectric substrate and *c* the speed of light in vacuum. The two identical two-ports C_1_ and C_2_ schematize the terminal adapters, while the two-ports L is the central load (not present in *strip-1*).

The application of a PMF modified both the parameters of the transmission lines (in a nonuniform way) and those of the two-ports, C_1_, C_2_, and L. As these variations were very small [7,10], the Born approximation held true, and mutual interactions (i.e., the variation of the response of a component not directly due to the PMF, but indirectly due to the variation of the responses of the other components) could be safely neglected, being of higher order. Hence, the overall variation of the SPs could be evaluated as a superposition of those of each single component of the TD. As already noted in the Introduction, this is a very important point, since it makes it the experimental results in Section 3 quite general, namely independent of the specific MW system wherein the component was inserted.

Accordingly, the variation of a generic SP due to the application of a PMF of intensity *H* is modeled as:

(1) $$\Delta s=s_{H}-s_{0}=\Delta s_{C1}+\Delta s_{C2}+\Delta s_{L}+\Delta s_{S},$$

where s_H_ and s_0_ are the SPs in presence and in absence of PMF, respectively, while Δs_C1_, Δs_C2_, Δs_L_ and Δs_S_ represent the variations of the SPs due to C_1_, C_2_, L, and the strip line, respectively, that one would measure if the PMF would be applied only on that component. As already noted, the contributions of Δs_C1_ and Δs_C2_ in (1) can be confidently neglected as compared to the other ones, as the intensity of the fringing PMF acting on such connectors is much lower than that acting on the other components.

**Figure 3:**
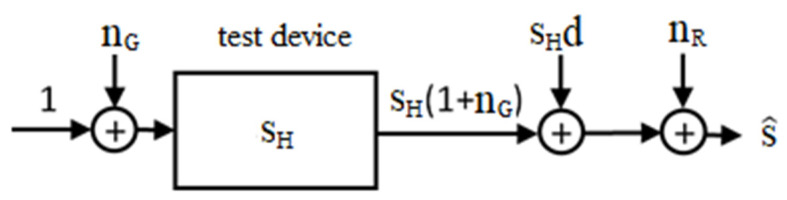
Scattering parameter (SP) measurement scheme.

Equation (1) expresses the variation of the SPs in the ideal case of absence of instrumental drift and measurement noise, which, however, are always present in any actual measurement and, therefore, must be adequately taken into account, with their level being comparable to Δs. To this end, we adopted the model depicted in Figure 3, where n_G_ and n_R_ denote the generator and receiver noises, respectively, d the instrumental drift, and $\hat{s}$ the SP actually measured (namely the noisy and drift-affected version of the term s_H_ in (1)). All the quantities in Figure 3 were normalized to the value of the input signal at the reference port, which was therefore assumed to be unitary.

The noise introduced by both the generator and the receiver was considered, assuming that both were modeled as zero mean additive white gaussian noise. This is a well-established assumption, confirmed by the measurement results reported in Section 3 showing that the characteristics and the mean square level of the noise are independent of the TD and of the measured SP. Of course, in Figure 3 the generator noise is multiplied times s_H_ since, like the incident wave, the noise propagates across the channel and, therefore, undergoes channel attenuation, represented by the amplitude of the corresponding SP, s_H_.

Concerning the instrumental drift, its precise characterization would require knowing the internal blocks of the instrument where it is generated and the causes for its generation. Therefore, here we adopted an operative approach, defining the drift as the relative difference between the SP measured in absence of noise and that measured in the ideal case (i.e., in absence of noise and drift). It is worth noting that, by its very definition, the drift could be inserted either at the transmitter side or, after multiplication by s_H_, at the receiver one, as shown in Figure 3. Here, we have opted for the latter choice because the experimental results reported in Section 3.2 and 3.3 show that drift depends on the amplitude of the received signal. This means that the drift is affected, in some way, by the received signal, so that it seems more appropriate to add it at the receiver side.

According to the scheme of Figure 3, $\hat{s}$, at a fixed microwave frequency, *f_µ_*, is given by:

(2) $$\hat{s}\left(f_{\mu },\underline{t}\right)=\left[1+n_{G}\left(\underline{t}\right)+d\left(\underline{t}\right)\right]s_{H}\left(f_{\mu },\underline{t}\right)+n_{R}\left(\underline{t}\right).$$

In (2), t is a vector of M entries, each being a time instant, *t_m_*, at which $\hat{s}$ was sampled. Specifically, *t_m_* = *m*Δ*t*, *m* = 0, …, M − 1, Δ*t* being the sampling step. As a result, also $\hat{s}$, s_H_, d, n_G_ and n_R_ are vectors of M entries each representing the value of that quantity measured at the time instant *t_m_*.

In the spectral domain (i.e., by performing an FFT of $\hat{s}$ and a proper zero frequency shift), (2) becomes:

(3) $$\hat{S}\left(f_{\mu },\underline{\nu }\right)=\left[1+N_{G}\left(\underline{\nu }\right)+D\left(\underline{\nu }\right)\right]⋇S_{H}\left(f_{\mu },\underline{\nu }\right)+N_{R}\left(\underline{\nu }\right),$$

where the symbol “⋇” denotes circular convolution while ν is a vector of M entries each representing the frequency, *ν_m_*, at which the spectrum of $\hat{s}$ was evaluated (*ν_m_* = *m*Δ*ν*, with Δ*ν* = (MΔ*t*)^−1^ and *m* = × (M − 1)/2, …, 0, …(M − 1)/2, in the case M is an odd integer). As a result, $\hat{s}$, S_H_, D, N_G_ and N_R_ are also vectors of M elements, each representing the value of that quantity at the frequency *ν_m_*.

In the case of a sinusoidal PMF, at frequency *ν_H_*, as in [11,16], S_H_(*f_µ_*, *ν*) is a sequence of discrete impulses concentrated at the frequencies *nν_H_*, with *n* being an integer number. In formulae:

(4) $$S_{H}\left(f_{\mu },\underline{\nu }\right)=\sum_{m=-\frac{M-1}{2}}^{\frac{M-1}{2}}s_{m}\left(f_{\mu }\right)\delta_{\nu_{m},n\nu_{H}}$$

where *δν_m_*,*nν_H_* is the Kronecker’s delta function and the coefficients s*_m_* are equal to the impulse amplitudes, for *m* = *nν_H_*/Δ*ν* (i.e., when *ν_m_* = *nν_H_*). Of course, in order to have *m* = *nν_H_*/Δ*ν*, one must set *ν_H_* = *q*Δ*ν*, being *q* a fixed integer number not larger than (M−1)/2n (in order to have at least the first *n* harmonics of S_H_ falling inside the analyzed band B = [−1/(2Δ*t*), 1/(2Δ*t*)]).

Since the application of the PMF only yields a small variation of the SPs, it follows that |s*_m_*| << |s_0_| *m* ≠ 0. Then, by noting that the drift and the noise levels are much lower than |s_0_|, (3) can be confidently approximated as:

(5) $$\hat{s}\left(f_{\mu },\underline{\nu }\right)=S_{H}\left(f_{\mu },\underline{\nu }\right)+s_{0}\left(f_{\mu }\right)\left(N_{G}\left(\underline{\nu }\right)+D\left(\underline{\nu }\right)\right)+N_{R}\left(\underline{\nu }\right).$$

Now, by assuming that n_G_ and n_R_ are uncorrelated, from (5) we have:

(6) <|S^|2>=<|SH+s0|D|2>+|s0|2σG2+σR2M≅|SH|2+|s0|2<|D|2>+2|s0|2Re<D>+|s0|2σG2+σR2M,

where the symbol “< >” denotes the (ensemble) average, σ_G_ and σ_R_ the standard deviations of n_G_ and n_R_, respectively (for the sake of brevity in (6), we have omitted the dependence on *f_µ_* and ν). Moreover, if we assume that, as is reasonable, the elements of the drift vector d(t) are stochastic variables with uniformly distributed phase and independent amplitude, we have Re<D> = 0, so that (6) becomes:

(7) $$<{\left|\hat{S}\right|}^{2}>={\left|S_{H}\right|}^{2}+{\left|s_{0}\right|}^{2}<{\left|D\right|}^{2}>+\frac{\left({\left|s_{0}\right|}^{2}\sigma_{G}^{2}+\sigma_{R}^{2}\right)}{M},$$

where:

(8) $${\left|S_{H}\right|}^{2}=\sum_{m=-\frac{M-1}{2}}^{\frac{M-1}{2}}{\left|s_{m}\right|}^{2}\delta_{\nu_{m},n\nu_{H}}.$$

Equation (7) is the relation we will exploit in the following to derive a design formula allowing us to establish the maximum PMF intensity that ensures a negligible spurious harmonic generation, as well as the PMF-modulation conditions under which the instrumental drift can be neglected. To this end, for each TD at hand, we need to experimentally characterize the behavior of the amplitudes of the different harmonic components in (8) vs the PMF amplitude, as well as the term <|D|^2^> in (7). The mean power spectra in (7) can be estimated through the ensemble average of the squared amplitude of the spectrum of a proper number of measurements of $\hat{s}$. The results will be presented and discussed in Section 3.

### 2.4. Experimental Setup

Since this characterization was originally meant for (but not limited to) the application to MNPs-enhanced MWI of breast cancer, the same set-up as [11] was considered in this study.

**Figure 4:**
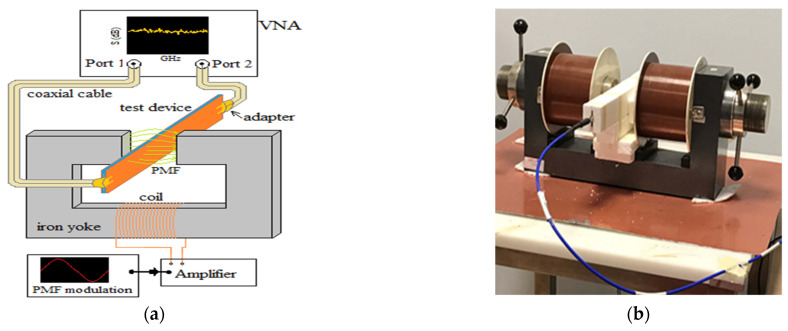
Overall measurement setup adopted for the magnetic test: (**a**) scheme; (**b**) a picture.

Figure 4 shows the overall experimental setup. As can be seen, the PMF was applied by inserting, in turn, one of the three TDs (sandwiched between two pieces of foam, 4 cm thickness, acting both as a spacer and protection to prevent damaging) between the poles of an electromagnet (EMU-75, from SES and Techno Instruments), with an air gap of 4 cm.

The electromagnet was fed by a power amplifier (Cerwin-Vega, CV-900) driven by a sinusoidal waveform generator, allowing one to generate a PMF with a peak amplitude up to 80 kA/m, within the set air gap aperture. The magnetic spurious effects were evaluated by measuring variations in the measured SPs of the TD due to the application of PMFs of different strengths. To this end, we employed a two-ports VNA (model Planar 809/1 from Copper Mountain), plugged to the TD through two sma coaxial cables.

The PMF was applied orthogonally to the strip and had a sinusoidal wave form with a modulation frequency, *ν_H_* = 5 Hz. This value was set in [11] in order to filter out the effects of the instrumental drifts, as well as variations of the measured signal due to the patient’s movement and vital activity. Analogously, as in [11], the SPs were measured at *f_µ_* = 2.37 GHz.

The IF bandwidth of the VNA was set equal to 100 Hz. For each measurement, we collected 10,001 samples sequentially, for an overall acquisition time of about 120 s per single time-sweep (about 12 ms per single acquisition). These values were a trade-off between the need to reduce, as much as was possible, the spectral noise increasing the number of acquisitions per time-sweep, while keeping the measurement time reasonable. For each time-sweep, the four SPs of the TD were acquired, and their spectrum was computed by means of an inverse FFT, thus preserving the amplitude of the modulation harmonics, while reducing the spectral noise. Specifically, a number of samples slightly lower than 10,001, equal to 9993 (by selecting samples from 9-th to 10,001-th), was exploited for the evaluation of the spectra, corresponding to a time sweep of 119.200 s in order to cover an integer number of periods of the applied sinusoidal PMF.

## 3. Results and Discussion

### 3.1. Assessment of the Microwave Response in Absence of Polarizing Magnetic Field

As a preliminary step, we have characterized the response of the three TDs over the frequency range of 2–3 GHz, which is the frequency band of main interest for MNPs-enhanced MWI of breast cancer [7].

**Figure 5:**
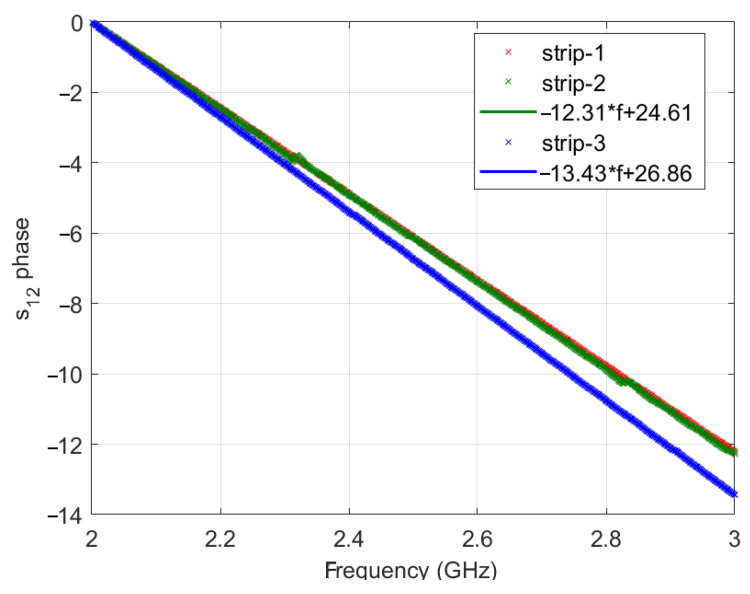
Phases of s_12_ vs frequency for the three TDs. The fitted frequency behavior is also shown.

The mean amplitudes and the standard deviations of the measured SPs are reported in Table 1, whereas the phases of s_12_ (s_21_) vs frequency (assumed equal to zero at 2 GHz) are shown in Figure 5.

The values reported in Table 1 and the linear behavior vs frequency of the s_12_ phase, say *ɸ*, observed in Figure 5 show that, as expected, all three TDs are well matched and behave as homogeneous and lossless transmission lines, characterized by an equivalent length *l_eq_* = *c*Δ*ɸ*/(2πΔ*f*) or equivalent permittivity ε*_eq_* = (*l_eq_*/*l*)^2^. For completeness, in Table 1, we also report the values of such equivalent parameters estimated by performing a linear fitting on the data in Figure 5. As expected, the equivalent permittivity in both cases is slightly lower than the nominal value *ε_s_* = 2.33 of the dielectric substrate of the strip. This is due to the presence of the sma-strip line adapters and the through, which are (only partially) filled by a dielectric material (PTFE disks supporting the inner conductor) characterized by a lower permittivity.

The above results ensure that, when evaluating spurious effects due to the application of a PMF, the same operating conditions hold in all the three cases.

### 3.2. Assessment of Spurious Magnetic Effects on Strip-1

Firstly, we investigated the effect of the PMF on *strip-1*. The aim is to assess the effects of the force exerted by the PMF on the MW conduction currents flowing in nonmagnetic conductors, such as copper, which typically employed in MW systems.

**Figure 6:**
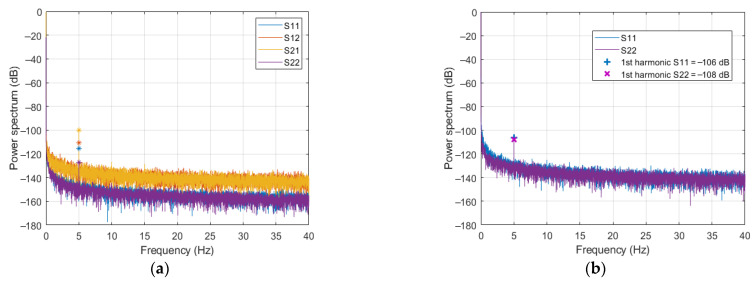
Power spectra of the SPs measured in the presence an applied sinusoidal polarizing magnetic field (PMF) of 80 kA/m: (**a**) *strip-1* plugged in to the VNA; (**b**) leaving the ports of the VNA floating.

The power spectra (i.e., the mean squared amplitude of the positive and negative frequency harmonics) of the SPs, measured at the reference ports of *strip-1*, in the presence of a PMF of (peak) amplitude *H* = 80 kA/m, are shown in Figure 6a.

From the figure, the following remarks can be outlined.

- As expected, in agreement with (5), all spectra show decreasing noisy amplitudes for *ν* ≠ *nν_H_* (with *n* natural number), representing the instrumental drift plus noise. Moreover, both drift and noise levels are different for reflection and transmission SPs. Specifically, those of s_11_ and s_22_ are about 20 dB lower than those of s_21_ and s_12_, which is just the ratio of the amplitudes of reflection and transmission SPs (see Table 1). This result is again in good agreement with (5)–(7), according to which both the drift and the generator noise are multiplied by the s_0_ of the corresponding SP. The receiver noise, which is independent of s_0_ and has a spectral level of about −163 dB, is masked by the generator noise.
- Figure 6a also shows that the drift, being slowly variable in time, has a spectrum confined to low frequencies. As already stressed in [11,16], this implies that the choice of the PMF modulation frequency, *ν_H_*, plays a crucial role in counteracting the effect of the instrumental drift. Of course, such choice depends on the employed measurement instrumentation, but also on the MW system being considered and on the amplitudes of the SPs being the drift multiplied by s_0_. This point will be discussed in Section 5.
- All the spectra exhibit a 1st order harmonic at the PMF modulation frequency *ν_H_* = 5 Hz, while no higher order harmonics, i.e., at *ν* = *nν_H_*, *n* > 1, are observed (at least above the drift and noise levels). As previously noted, a possible cause of such harmonic components could be the modulation of the currents flowing in the conductors of the strip, due to the force exerted on the charge carriers by the PMF. This force translates into an anisotropic modulation of the conductivity that, at the main order, is proportional to the applied PMF. However, this hypothesis is not consistent with the significant difference observed in Figure 6a among the amplitudes of the harmonics of the four SPs. Indeed, while a difference is possible between reflection and transmission SPs, this cannot happen between s_12_ and s_21_, or between s_11_ and s_22_, due to the symmetry of the experimental setup, which makes an exchange between ports 1 and 2 equivalent to a rotation of 180 degrees of the full device. This suggests that the observed first harmonic is not ascribable to the PMF modulation of the conduction currents, or, more generally, to an effect of the PMF on the TD, but more likely to an influence of the PMF on the VNA. To check this hypothesis, we performed an additional measurement in the same PMF conditions, but disconnected the TD from the VNA and left the ports of the VNA floating. The results are shown in Figure 6b, where only the spectra of s_11_ and s_22_ are reported. As can be seen, a first harmonic is still present, and its amplitude is even larger than the one measured in the presence of *strip-1*. Such a result makes us confidently conclude that the observed first harmonics is prevalently an effect of the fringing PMF on the VNA (possibly on its inner electronics).

From the above investigation on strip-1, one can conclude that no detectable spurious harmonics arise from the PMF modulation of the conduction currents, hence, from nonmagnetic metals (or, in general, conductors), at least for PMF amplitudes smaller than 80 kA/m. Conversely, a spurious harmonic at the same frequency *ν_H_* of the applied PMF is observed, due to the interaction between the PMF and the VNA. Of course, such a harmonic can be simply filtered out if the useful signal is characterized by a different spectral content, as it happens in an MNPs-enhanced MWI, where the useful signal has a frequency of 2*ν_H_*. Moreover, it can be lowered by moving the VNA further away from the electromagnet or shielding it from the PMF.

### 3.3. Assessment of Spurious Magnetic Effects on Strip-2 and Strip-3

The aim of this experiment is to assess the spurious signal due to the central elements of the TDs, wherein magnetic materials can be present. Such a signal is at the same frequency as the useful signal (i.e., the one generated by the MNP-loaded tumor). Hence, it cannot be simply filtered out and must be counteracted by limiting the amplitude of the PMF acting on the relevant elements.

**Figure 7:**
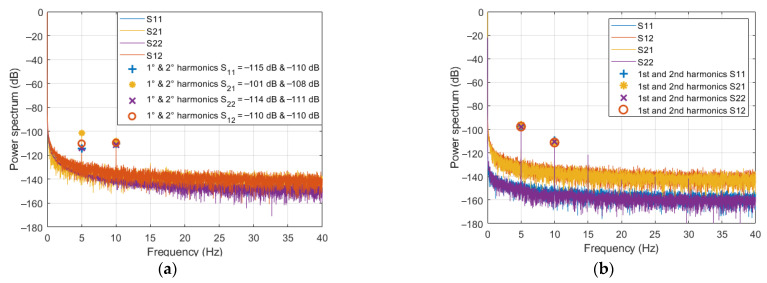
Power spectra of the measured SPs in the presence of an applied sinusoidal (*ν_H_* = 5 Hz) PMF: (**a**) *strip-2* and *H* = 80 kA/m; (**b**) *strip-3* and *H* = 10 kA/m.

The experiment consisted in measuring the four SPs for different PMF amplitudes, *H*. Specifically, *H* has been set equal to 20, 30, 40, 60, 80 kA/m for *strip-2* and equal to 4, 6, 8, 10 kA/m for *strip-3*. Lower PMF amplitudes have been considered for *strip-3*, since a stronger response is expected in this case due to a larger amount of magnetic material present in the TD. For each strip and PMF amplitude, five independent measurements were performed (each measurement providing the 9993 samples of the four SPs), in order to allow an estimate of the mean values and standard deviations of the quantities of interest. As an example, Figure 7 shows the power spectra of the SPs relative to one of the measurements performed on *strip-2* (panel a) and *strip-3* (panel b) at the maximal amplitudes of the applied PMF, namely *H* = 80 kA/m for *strip-2* and *H* = 10 kA/m for *strip-3*.

From the figure, the following remarks can be outlined.

- For both *strip-2* and *strip-3*, a first order harmonic component is present in all the spectra. In *strip-2*, the amplitudes of such harmonics are comparable to those observed for *strip-1*, and exhibit the same behavior, i.e., they are different for the four SPs. On the other side, in the case of *strip-3*, these harmonics have quite the same amplitude for all the SPs, which is larger than that observed for *strip-1* and *strip-2*, despite the applied PMF being 8 times lower. These observations suggest that the central welding in *strip-2* does not significantly respond to the applied PMF, so that the first harmonic is still essentially due to an effect of the PMF on the VNA. Conversely, in the case of *strip-3*, the first harmonic (much larger, if referred to the same PMF amplitude) appears to be prevalently due to the central through.
- A second order harmonic is also present, with an amplitude of about −110 dB for all the SPs in both cases. According to the results in Section 3.2, this harmonic contribution cannot be ascribed to the strip-line or to the VNA, but to the central loads, since *strip-2* and *strip-3* differ from *strip-1* only for the central load, wherein magnetic materials are likely to be present. While this was expected for the through and the connectors, it is somewhat surprising for the welding. Either way, the second harmonic generated by the welding is much lower, confirming that the connectors are the components that, more than any others, should be kept away from the PMF to prevent spurious magnetic effects.
- For *strip-3*, one can also observe a third order harmonic not present in the SPs of *strip-2*. Again, such a harmonic exhibits the same amplitude of about −122 dB for all four SPs. A possible explanation of this outcome is reported in the Appendix A.

**Figure 8:**
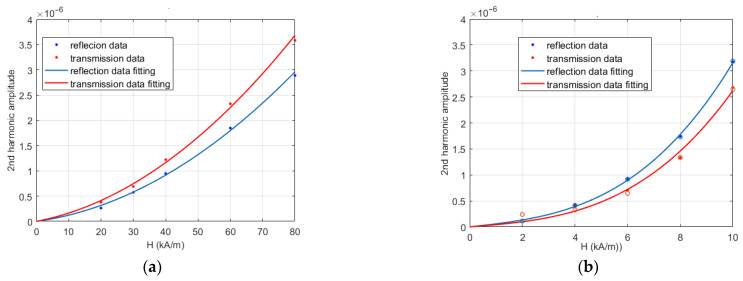
Mean values vs PMF of reflection (blue dots) and transmission (red dots) of 2nd harmonics’ amplitudes and corresponding fitting curves. (**a**) *strip-2*; (**b**) *strip-3*. The red circles denote additional measurements performed to check the derived model.

Figure 8 reports (in linear scale) the mean amplitudes of the second harmonic (which is the one of interest in MNPs-enhanced MWI) of the reflection (blue dots) and transmission (red dots) SPs, for each of the considered values of *H* and for *strip-2* (panel a) and *strip-3* (panel b), respectively. Each point represents the mean square value of the amplitudes of the corresponding two harmonics (at ± 2*ν_H_*), averaged over the five measurements. Being very small, in the order of 10^−7^, the standard deviations have not been reported.

From Figure 8, one can note that, in both cases, the percentage difference between the reflection and transmission harmonic amplitudes is small, confirming their already noted substantial equality. Figure 8 also confirms that the second harmonic generation is much stronger in the case of *strip-3*, i.e., for the through connector.

To determine the behavior of the second harmonic’s amplitude as a function of the PMF amplitude *H*, which is necessary for deriving design guidelines, we made a mean square fitting of the data in Figure 8 with a homogeneous polynomial. To minimize the number of free parameters, we added just another term to the linear one. In particular, a quadratic term is considered for *strip-2*, while a cubic term is used for *strip-3*, as the latter clearly exhibits a larger departure from linearity. In this way, we obtained the following expressions for the mean values of the reflection and transmission harmonics amplitudes, say *A_r_* and *A_t_*:

(9) $$<A_{r}>=8.77\times 10^{-9}H\left(1+0.0402H\right),$$

(10) $$<A_{t}>=12.2\times 10^{-9}H\left(1+0.0347H\right),$$

for *strip-2*, and:

(11) $$<A_{r}>=5.63\times 10^{-8}H\left(1+0.046H^{2}\right),$$

(12) $$<A_{t}>=4.05\times 10^{-8}H\left(1+0.055H^{2}\right),$$

for *strip-3*. The corresponding curves are reported in Figure 8 as blue and red lines, respectively, and show the good quality of these fittings.

To check the predictive power of the Formulae (9)–(12), we performed a further set of (single) measurements on *strip-3*, also adding a measurement at *H* = 2 kA/m. The corresponding second harmonics amplitudes are reported as circles in Figure 8. As it can be seen, the agreement is excellent: even the transmission harmonic at 2 kA/m is predicted reasonably well, notwithstanding its amplitude, which is comparable to the drift spectral level (see Figure 6a).

### 3.4. Assessment of the Instrumental Drift

Regarding the drift, according to (7), apart from the spectral components at *nν_H_*, *n ≥* 0, the average power spectrum of the corresponding SP, normalized to |s_0_|^2^, is equal to the average drift power spectrum, plus a (constant) mean square noise term.

**Figure 9:**
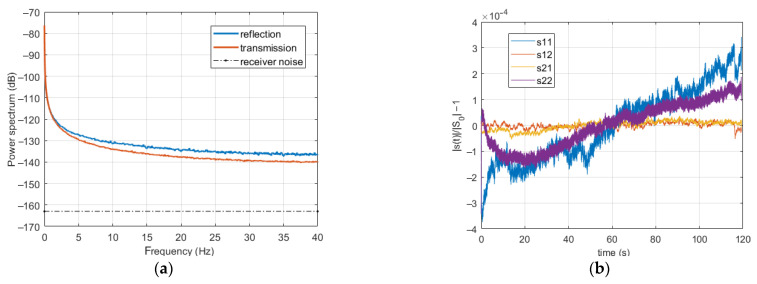
Average drift plus generator noise power spectra (panel **a**) and normalized SPs drift (panel **b**).

For estimating the average normalized reflection and transmission power spectra of the drift, we exploited all the measurements performed on *strip-3*. In particular, to further reduce the variance of the residual noise, we also smoothed the power spectra by performing a sliding window average, with a window width of 25 points. After subtraction of the term related to the receiver noise, we obtained the reflection and transmission drift plus generator noise average normalized power spectra shown in Figure 9a. In the same figure, the (spectral) mean square value of the receiver noise is also reported.

The spectra initially decrease very rapidly, then they slowly approach a constant level much higher than that of the receiver noise. The reflection spectrum is higher than the transmission spectrum, with a limit difference of about 1.4 × 10^−14^. As the contributions of the generator noise cancel out, this shows that the reflection and transmission spectra of the drift are different.

To understand the cause of such a difference, let us observe the time domain behavior of the normalized SPs. An enlightening example (relative to one of the measurements on *strip-3*, with *H* = 8 kA/m) is reported in Figure 9b, showing the amplitudes of the normalized SPs, after subtraction of 1 and a sliding window average, to reduce the noise. As can be seen, the reflection SPs exhibit a much more significant drift, and, more importantly, a much larger difference between the final and initial values, implying a stronger asymptotic spectral amplitude, slowly decaying as 1/*ν*. Even if, in other cases, the difference between the drifts of reflection and transmission SPs can be smaller, on average, it is significant, and explains why the reflection and transmission average drift power spectra are not the same. As might have been expected, this implies that the drift is affected by the level of the signal impinging on the receiver, and increases as this level, namely |s_0_|, decreases. However, the increase in drift amplitude is smaller than the decrease in the SP amplitude, so that the term |s_0_|^2^ < |D|^2^> in (7) decreases by decreasing |s_0_|.

The relevance of this point on the development of design guidelines will be discussed in the next section.

## 4. Design Guidelines

By exploiting the experimental results presented in the previous sections, we can now derive the conditions on the PMF intensity under which one can safely neglect the spurious harmonics generation arising from tin welding and connectors of any n-ports MW system, subject to an applied sinusoidal PMF (as the one in [11]), as well as the conditions on the PMF modulation frequency under which one can neglect the effect of instrumental drift.

Let us first investigate the maximum PMF intensity allowed on tin welding or connectors, ensuring that the spurious harmonics can be neglected. We assume that such components are placed before the ports of the MW system (when looking toward the system), as this is what usually happens in practice (in particular, for the system in [11]). As pointed out in Section 2.3, since the overall affect is obtained by superposition, we can analyze the individual contributions separately.

To determine the maximum PMF intensity, we can exploit (7), where the term |S_H_|^2^ now indicates the squared amplitude of one of the spurious harmonics of interest (in our case, the reflection or transmission 2nd harmonic at *ν* = 10 Hz) generated by the component at hand (solder or connector) and the term s_0_ one of the entries, say Σ*_ij_* (*i,j* = 1, …, n), of the scattering matrix of the (unperturbed) n-port MW system. In the case of a solder, the amplitudes of the spurious harmonics generated under a unit incident voltage and a PMF of intensity *H*, are provided by (9) and (10); for a male–female couple of connectors, a pessimistic, hence conservative, estimate is provided by (11) and (12), which refer to a component consisting of two male connectors plus a through.

With reference to the generic couple of ports *ij*, let us denote with *R_ii_*, *R_jj_* and *T_ij_* the reflection and transmission spurious harmonics generated by the component at hand, placed before the port *i* of the (n-port) MW system under test.

By approximating at the leading order, we have:

(13) $$R_{ii}=<A_{,r}>exp\left(j\varphi_{r}\right)+<A_{t}>exp\left(j\varphi_{t}\right)\Sigma_{ii}$$

(14) $$R_{jj}=\Sigma_{ji}<A_{r}>exp\left(j\varphi_{r}\right)\Sigma_{ij},$$

(15) $$T_{ij}=<A_{t}>exp\left(j\varphi_{t}\right)\Sigma_{ij}=T_{ij}$$

*φ_r_* and *φ_t_* indicating the (unknown) phases of the harmonics generated by the component alone (inclusive of the propagation phase shift up to the proper reference section).

Assuming that the MW system is reasonably well matched, the second term of the right-hand side of (13) can be safely neglected, so that the unknown phase factors become inessential for the evaluation of |S_H_|^2^ in (7), which can be made exploiting (9)–(12).

Moreover, comparison between (13) and (14) shows that, being |Σ*_ji_*| = |Σ*_ij_*| < 1, due to the losses, the most critical condition is just that of the port wherein the component is located, for which we have |S_H_|^2^ = <*A_r_*>^2^ for the reflection harmonic and |S_H_|^2^ = <*A_t_*>^2^|Σ*_ij_*|^2^ for the transmission one. Inserting these expressions in |S_H_|^2^ in (7), we obtain the following conditions on the PMF amplitude *H*, ensuring that the spurious harmonics can be neglected with respect to drift and noise. For a reflection harmonic we have:

(16) $$<A_{r}{\left(H\right)>}^{2}<{\left|\Sigma_{ii}\right|}^{2}\left(\frac{<{\left|D_{ii}\right|}^{2}>+\sigma_{G}^{2}}{M}\right)+\frac{1}{M}\sigma_{R}^{2},$$

where D*_ii_* denotes the drift of the reflection coefficient measured at the *i*-th port.

For the transmission harmonic we obtain:

(17) <At(H)>2<(<|Dij|2>+σG2M)+1M(σR|Σij|)2,

where D*_ij_* denotes the drift of the transmission coefficient measured at the couple of ports *ij*.

As discussed at the end of the previous section, the drift power spectrum increases when the amplitude of the corresponding SP decreases, but less rapidly. Hence, the first term on the right-hand side of (16) decreases with |Σ*_ii_*|, so that it is not larger than the red curve in Figure 9a, which is the drift power spectrum relative to an SP with an amplitude slightly smaller than one. The opposite occurs for the first term on the right-hand side of (17). As the second term is also not smaller than the corresponding one of (16), we conclude that the right-hand side of (17) is always not smaller than that of (16). Because <*A_r_*> and <*A_t_*> are almost equal (see Figure 8), this implies that transmission measurements are less vulnerable to spurious harmonics, particularly when the MW system under test works in the presence of lossy media, as happens in MWI and, more generally, in the case of biomedical applications. From a physical point of view, this is quite an intuitive result. Indeed, while in reflection mode, the spurious harmonic does not undergo any attenuation; in transmission mode, the same harmonic undergoes the attenuation characterizing the path connecting the ports *ij*, which can be quite significant.

However, it must be noted that the level of the useful harmonic generated by the MW system under test depends, in a nonlinear way, on the amplitude of the PMF acting on the target and on its position, which strongly affect the relative amplitude of the transmission and reflection harmonics. As such, when exploiting (16) and (17) for determining the maximum allowed value of *H*, one must take into account the configuration and operative conditions of the MW system under test, making reference to the worst case.

Coming to the drift, if one wants to achieve the detection limits set by the receiver noise, according to (7), the PMF modulation frequency *ν_H_* must be such that:

(18) $${\left|\Sigma_{ij}\right|}^{2}\left(<{\left|D_{ij}\left(2\upsilon_{H}\right)\right|}^{2}>+\frac{\sigma_{G}^{2}}{M}\right)<\frac{\sigma_{R}^{2}}{M}$$

Since the drift spectrum in (18) decreases with frequency, it is obviously convenient to set *ν_H_* as high as possible to counteract its influence. However, because, after the initial very rapid drop, the decrease becomes slow and due to the presence of the generator noise term in (18), an increase of the modulation frequency above a certain value becomes useless in practice. Such a limit obviously depends on both the characteristics of the VNA and the value of |Σ*_ij_*| that determine the frequency behavior of the left-hand member of (18). This latter is just the (not normalized) drift plus generator noise power spectrum, whose amplitude, as seen above with reference to (16), decreases with the amplitude of the SP |Σ*_ij_*|. Again, in the case of an MW system working in presence of lossy media, transmission measurements prove to be more convenient, also from a reduction in the drift influence.

However, it must be stressed that it could not be possible to achieve the receiver noise limit. For instance, from Figure 9, we observe that, in the case of our TDs, condition (18) cannot be satisfied at the second harmonic frequency of 2*ν_H_* = 10 Hz if |Σ*_ij_*| is higher than −30 dB.

### Application to the MNPs-Enhanced MW Detection of Cancer

As an example of application and validation of the above design formulas, let us particularize them to the case of MNPs-enhanced MW detection of cancer by exploiting the experimental results reported in [11], which were obtained by using simplified, but realistic, breast phantoms. As already stated, the operative conditions, the measurement apparatus, and the settings were the same as those adopted in this paper, with a PMF amplitude equal to 40 kA/m.

The measured amplitudes of the transmission SP of the phantoms alone were between −47 and −53 dB, depending on their composition. As already noted, the (mean) spectral level of the receiver noise was −163 dB, while that of the normalized drift plus generator noise, at 2*ν_H_* = 10 Hz, was around −113 dB. Hence, condition (18) is not satisfied for just 3 dB, which is well inside the noise power fluctuations, meaning that the drift is practically merged in the receiver noise and, thus, is in agreement with the results reported in [11].

In those experiments, the MNPs were located at the center of the phantom, at equal distances from the transmitting and receiving antennas. Accordingly, equal reflection and transmission harmonics are almost equal, supporting the exploitation of transmission harmonics to tame spurious effects.

In particular, to reduce the effect of spurious harmonics to the receiver noise level, even in the less favorable case (maximum level of |Σ_12_| = −47 dB), we must ensure a second spurious harmonic level lower than about −163 + 47 = −116 dB ≈ 1.5 × 10^−6^. According to Figure 8, this means that *H* must be lower than 45 kA/m for the case of a solder, and 8 kA/m for a connector. To validate this estimate, we measured the PMF intensity in correspondence with the connectors of the antennas mounted on the phantom used in [11], which turned out to be equal to about 9 kA/m. By also considering that the connectors of the cables plugging the antenna to the VNA contribute to the second harmonic generation, this correlates quite well with the presence of a second harmonic about 9 dB above the noise level, in absence of MNPs, observed in all the three phantoms investigated in [11].

The simplest solution to reducing the spurious harmonic amplitude below the receiver noise (thus increasing the detection capability of the system) is to move the connectors away from the poles of the electromagnet, exploiting the fast decay of the fringing field. Another, less easy to implement, possibility is to employ connectors free of magnetic materials, hence manufacture ad hoc connectors for the application at hand.

## 5. Conclusions

In this paper, we have investigated the spurious effects that the application of a PMF, needed to implement MNPs-enhanced MWI of cancer, can produce on components and parts typically composing an MW system, due to its action on both the conduction currents and magnetic materials possibly hidden in such components. This has been performed by exploiting specifically designed, simple yet effective, TDs, which, however, include all those components of an MW system (such as printed metallic elements, welding and connectors) whose response can be affected by a PMF. The use of the ad-hoc designed TDs relies on the fact that the spurious signals, due to each component/part of a MW system, do not affect each other (since their mutual interactions are negligible). Accordingly, one can characterize the response of the single components separately (i.e., not necessarily as a whole) by including them in a TD properly meant for this purpose.

Furthermore, we have analyzed the effects of instrumental drift, which can represent a significant source of disturbance when, as it happens in the case of MNPs enhanced breast cancer MW detection, many repeated measurements are needed in order to reduce the noise and detect very low-level signals.

The experimental investigation has shown that no appreciable effects arise from the modulation of the conduction currents, at least for PMF intensity up to 80 kA/m. This also confirms that no appreciable effects can arise from the presence of phantoms (mimicking anatomical parts of the body) in the experimental setup.

Unexpectedly, an appreciable effect on the measurement instrument (a VNA in our case) was observed at the same frequency of the applied sinusoidal PMF, possibly due to an interaction between the PMF and the inner VNA electronics. Of course, this effect can be reduced either by shielding the measurement device or simply by moving it away from the PMF source. However, in all applications where the useful signal has a different spectral content, as it is the case of MNP enhanced MW cancer detection, the first order harmonic, due to the instrument, can be simply erased by filtering the relevant frequency.

Conversely, a remarkable second order harmonic, arising from welding and connectors, has been observed, which likely due to the presence of hidden magnetic materials. Such a harmonic is much more pronounced for connectors, being about eight times larger and characterized by a faster growth with the PMF amplitude. Therefore, the connectors (and, possibly, the terminal tracts of the cables connecting the MW system to the measurement ones) represent the most critical MW components, particularly in those applications wherein the useful signal is just the second harmonic, as happens when MNPs (or other dispersed magnetic materials) are exploited as modulable contrast agents for diagnostic or therapeutic [17] purposes.

Exploiting the results of the experimental investigation, we have derived design guidelines for the choices of proper PMF amplitude and modulation frequency that allow for taming of the spurious magnetic effects and drift. The analysis has highlighted that, in the case of an MW system working in the presence of lossy media, transmission measurements are generally less vulnerable to both the spurious harmonics and drift; therefore, they should be preferred to reflection measurements.

It is worth noting that the substantial absence of effects due to the PMF modulation of the conduction currents would suggest using components free of magnetic material in order to counteract spurious magnetic effects. However, while this solution is effective and simple from the standpoint of the design of the system, it has the drawback of being less easy (and cheap) to implement, as it requires the adoption of components free from magnetic materials, hence, manufactured ad hoc for the application at hand. Accordingly, the proposed design guidelines represent a suitable trade-off between the need to keep the spurious magnetic effects low and the need to use standard microwave components for the implementation of the MW system.

As an assessment of the validity of the outcomes of this study, the obtained design formulae have been applied to the experimental results in [11], showing that the spurious effects observed for the MWI system in [11] are well predicted. In addition, this validation confirms that, as foreseen, the entity of the spurious signal is not affected by the presence of the phantom and by the overall structure of the system under test.

Last but not least, while the overall experimental setup (i.e., TDs, PMF modulation type and frequency, microwave working frequency, measurement instrument, and so on) adopted in this study is meant for the MWI system in [11], the approach adopted to derive the formulae (16)–(18) is completely general. In other words, the design Equations (16)–(18) hold for (and can be applied to) any system, provided the values of the quantities appearing in the equations (i.e., harmonic amplitude, scattering parameter amplitude, instrumental drift, noise level) are properly replaced with those relevant to the specific system under test. Of course, the last consideration is not limited to the application of MNPs-enhanced MW diagnostic or therapy, but it can be extended to all those applications where a coherent modulation of the response of the useful signal is adopted in order to increase the signal-to-noise ratio.

## Figures and Tables

**Table 1:** Mean amplitude and standard deviation of the SPs of the three TDs, over the band of 2–3 GHz. Estimated equivalent length and relative electric permittivity.

TD	|s_11_|	|s_22_|	|s_12_| [|s_21_|]	*l_eq_* [mm]	ε*_eq_*
*strip-1*	0.071 ± 0.025	0.067 ± 0.024	0.928 ± 0.090	584	2.03
*strip-2*	0.119 ± 0.051	0.114 ± 0.067	0.906 ± 0.027	588	2.06
*strip-3*	0.076 ± 0.035	0.072 ± 0.029	0.918 ± 0.009	641	2.12

**Figure A1:**
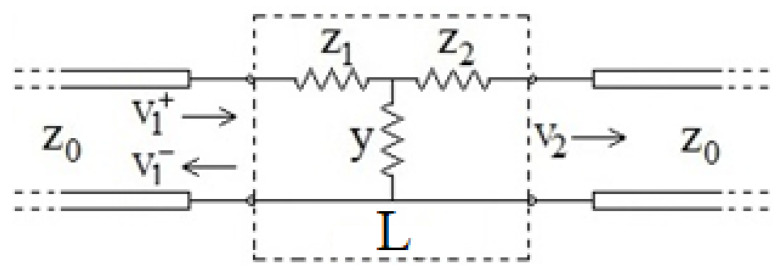
Schematization of the central load, L.

## Abbreviations

The following abbreviations are used in this manuscript.

MNP	Magnetic Nanoparticle
MWI	MicroWave Imgning
PMF	Polarizing Magnetic Field
TD	Test device
SP	Scattering parameter
VNA	Vector Network Analyzer

## Appendix A

In this Appendix, we provide a possible explanation of the observed substantial equality of the amplitudes of the harmonics of the same order generated by the central load L (sold and trough, respectively).

To this end, let us refer to the T-shape equivalent network representing the (PMF dependent) element L, reported in Figure A1, wherein the impedances are normalized to the characteristic impedance of the strip-line and the reference ports are assumed shifted at the center of L (which does not change the amplitude of its scattering parameters).

Because the central loads are very well adapted (see Section 3) and, with the adopted reference, all the components of the equivalent network would be equal to zero in the case of perfect matching, their absolute values are much less than one. Accordingly, in the evaluation of the scattering parameters, we can safely limit ourselves to the terms of first order in z_1,2_ and y. In such a way, by straightforward computations, we obtain:

(A1) $$s_{11\;}=s_{22\;}=\left(z-y\right)/2\;,$$

(A2) $$s_{12\;}=s_{21\;}=1-\left(z+y\right)/2,$$

wherein z *=* (z_1_ + z_2_).

Equations (A1) and (A2) show that, to obtain approximately equal amplitudes of the reflection and transmission harmonics, the variation of the network parameters, say *δz* and *δy*, induced by the PMF, must be such that:

(A3) |δz−δy| ≈|δz+δy|.

Now, physically, z is related to the conductivity and the magnetic properties of conductors within the central load, while y is related to the permittivity of the dielectric. Therefore, it is quite natural to assume that the magnetic PMF mainly affects z, thus making *δz* significantly larger than *δy*, which implies (A3). Note explicitly that, depending on the phase shift between *δz* and *δy*, the amplitude of the reflection harmonics can be smaller (as for *strip-2*) or larger (as for *strip-3*) than the transmission ones.
